# Supplementary material for: Is breast arterial calcification associated with coronary artery disease?—A systematic review and meta-analysis
Source: PLoS One. 2020 Jul 28;15(7):e0236598. doi: 10.1371/journal.pone.0236598 (PMC7386618; doi:10.1371/journal.pone.0236598)
Supplement: S1 Table — * Asterisk used to capture multiple word endings (e.g. calcifying, calcification). (DOCX) [file pone.0236598.s001.docx]

| **Steps** | **MeSH headings and key words** |
| --- | --- |
| 1 | Breast OR mamma OR mammary OR mammograph* OR intramammary |
| 2 | Vessel OR vessels OR artery OR arterial OR arteries OR vascular |
| 3 | Calcif* OR scleros* or calcinos* or calcium |
| 4 | 1 AND 2 |
| 5 | 3 AND 4 |

* Asterisk used to capture multiple word endings (e.g. calcifying, calcification)
