# Supplementary material for: Is breast arterial calcification associated with coronary artery disease?—A systematic review and meta-analysis
Source: PLoS One. 2020 Jul 28;15(7):e0236598. doi: 10.1371/journal.pone.0236598 (PMC7386618; doi:10.1371/journal.pone.0236598)
Supplement: S2 Table — 1: representativeness of cohort (a—truly representative of the community; b—somewhat representative of the community; c—selected group; d—no description), 2: selection of non-exposed cohort (a—drawn from the same community as the exposed cohort; b—drawn from a different source; c—no description), 3: ascertainment of exposure (a—secure record or independent blind assessment; b—single blinded assessment; c—unblinded assessment; d—no description), 4: demonstration that outcome of interest was not present at the start of study (a—yes; b—no), 5: comparability/controlling for confounders (a—study controls for age; b—study controls for any additional factor), 6: assessment of outcome (a—independent blind assessment; b—record linkage; c—self report; d—no description), 7: duration of follow up (a—yes; b—no), 8: adequacy of follow up (a—complete follow up; b—subjects lost to follow up unlikely to introduce bias where >80% were followed up or description was provided of those lost; c—follow up rate <80% and no description of those lost; d—no statement), *: stars given for each question; x: answer marked for each question, +: good, ±: moderate. (DOCX) [file pone.0236598.s002.docx]

| **Author** | **Year** | **Selection** | | | | | | | | | | | | | **Comparability** | | **Outcome** | | | | | | | | | | **Total** | **Overall** |
| --- | --- | --- | --- | --- | --- | --- | --- | --- | --- | --- | --- | --- | --- | --- | --- | --- | --- | --- | --- | --- | --- | --- | --- | --- | --- | --- | --- | --- |
|  |  | **1** | | | | **2** | | | **3** | | | | **4** | | **5** | | **6** | | | | **7** | | **8** | | | |  |  |
|  |  | **a**  ***** | **b**  ***** | **c** | **d** | **a**  ***** | **b** | **c** | **a**  ***** | **b**  ***** | **c** | **d** | **a**  ***** | **b** | **a**  ***** | **b**  ***** | **a**  ***** | **b**  ***** | **c** | **d** | **a**  ***** | **b** | **a**  ***** | **b**  ***** | **c** | **d** | * |  |
| Soran (65) | 2014 | - | - | x | - | x | - | - | x | - | - | - | - | x | - | - | - | - | x | - | x | - | - | x | - | - | 4 | - |
| Kelly (47) | 2018 | - | - | x | - | x | - | - | x | - | - | - | - | x | x | x | - | - | - | x | x | - | x | - | - | - | 6 | ± |
| Abou-Hassan (62) | 2015 | - | - | x | - | x | - | - | x | - | - | - | - | x | x | x | - | x | - | - | x | - | x | - | - | - | 7 | ± |
| Schnatz (25) | 2011 | x | - | - | - | x | - | - | x | - | - | - | x | - | x | - | - | - | x | - | x | - | - | x | - | - | 7 | + |
| Iribarren (18) | 2004 | - | x | - | - | x | - | - | x | - | - | - | x | - | x | x | - | x | - | - | x | - | x | - | - | - | 9 | + |

1: representativeness of cohort (a – truly representative of the community; b – somewhat representative of the community; c – selected group; d – no description), 2: selection of non-exposed cohort (a – drawn from the same community as the exposed cohort; b – drawn from a different source; c – no description), 3: ascertainment of exposure (a – secure record or independent blind assessment; b – single blinded assessment; c – unblinded assessment; d – no description) , 4: demonstration that outcome of interest was not present at the start of study (a – yes; b – no), 5: comparability/controlling for confounders (a – study controls for age; b – study controls for any additional factor), 6: assessment of outcome (a – independent blind assessment; b – record linkage; c – self report; d – no description), 7: duration of follow up (a – yes; b – no), 8: adequacy of follow up (a – complete follow up; b – subjects lost to follow up unlikely to introduce bias where >80% were followed up or description was provided of those lost; c – follow up rate <80% and no description of those lost; d – no statement), *: stars given for each question; x: answer marked for each question, +: good, ±: moderate
