# Supplementary material for: Is breast arterial calcification associated with coronary artery disease?—A systematic review and meta-analysis
Source: PLoS One. 2020 Jul 28;15(7):e0236598. doi: 10.1371/journal.pone.0236598 (PMC7386618; doi:10.1371/journal.pone.0236598)
Supplement: S3 Table — 1: case definition (a—yes with independent validation; b—yes with record linkage, self report, non-independent validation; c—no description), 2: representativeness of cases (a—consecutive or obviously representative series of cases; b—potential selection bias or not stated), 3: selection of control (a—community controls; b—hospital controls; c—no description), 4: definition of control (a—no history of disease/endpoint; b—no description), 5: comparability/adjusting for confounders (a—controls for age; b—controls for other additional factors), 6: ascertainment of exposure (a—secure record or two blinded investigators; b—structured interview where blinded to case/control status or one blinded investigator; c—not blinded; d—written self report or medical record only; e—no description), 7: same method of ascertainment of exposure for cases and controls (a—yes; b—no), 8: non response rates (a—same rate for both groups; b—non respondents described; c—rate different and no designation), *: stars given for each question; x: answer marked for each question; +: good, ±: moderate. (DOCX) [file pone.0236598.s003.docx]

| **Author** | **Year** | **Selection** | | | | | | | | | | **Comparability** | | **Exposure** | | | | | | | | | | **Total** | **Overall** |
| --- | --- | --- | --- | --- | --- | --- | --- | --- | --- | --- | --- | --- | --- | --- | --- | --- | --- | --- | --- | --- | --- | --- | --- | --- | --- |
|  |  | **1** | | | **2** | | **3** | | | **4** | | **5** | | **6** | | | | | **7** | | **8** | | |  |  |
|  |  | **a**  ***** | **b** | **c** | **a**  ***** | **b** | **a**  ***** | **b** | **c** | **a**  ***** | **b** | **a**  ***** | **b**  ***** | **a**  ***** | **b**  ***** | **c** | **d** | **e** | **a**  ***** | **b** | **a**  ***** | **b** | **c** | ***** |  |
| Atci (54) | 2015 | - | x | - | - | x | - | - | x | x | - | - | - | - | - | - | - | x | x | - | x | - | - | 3 | - |
| Yagtu (33) | 2015 | - | x | - | - | x | - | - | x | - | x | x | - | - | - | x | - | - | x | - | x | - | - | 3 | - |
| Hanafi (29) | 2018 | x | - | - | x | - | - | x | - | - | x | - | - | - | - | - | x | - | x | - | x | - | - | 4 | - |
| Pidal (22) | 2009 | x | - | - | - | x | - | - | x | x | - | - | - | - | - | - | x | - | x | - | x | - | - | 4 | - |
| Sankaran (61) | 2019 | - | x | - | - | x | x | - | - | x | - | - | - | - | - | - | x | - | x | - | x | - | - | 4 | - |
| Dale (I) (60) | 2010 | - | x | - | - | x | x | - | - | x | - | x | - | - | - | x | - | - | x | - | x | - | - | 5 | ± |
| Kosovic (28) | 2015 | x | - | - | - | x | x | - | - | x | - | - | - | - | - | - | x | - | x | - | x | - | - | 5 | ± |
| Bae (31) | 2013 | - | x | - | - | x | x | - | - | x | - | - | x | x | - | - | - | - | x | - | x | - | - | 6 | ± |
| Schmitt (59) | 1985 | - | x | - | - | x | x | - | - | x | - | x | - | - | x | - | - | - | x | - | x | - | - | 6 | ± |
| Wada (64) | 2012 | x | - | - | x | - | x | - | - | x | - | x | - | - | - | x | - | - | x | - | - | x | - | 6 | + |
| Yildiz (III) (51) | 2008 | - | x | - | x | - | x | - | - | x | - | - | x | - | - | - | x | - | x | - | x | - | - | 6 | + |
| Yildiz (IV) (67) | 2016 | x | - | - | - | x | x | - | - | x | - | - | - | - | x | - | - | - | x | - | x | - | - | 6 | + |
| Yildiz (I) (70) | 2018 | - | x | - | - | x | x | - | - | x | - | x | x | - | - | - | x | - | x | - | x | - | - | 6 | ± |
| Sedighi (24) | 2011 | - | x | - | x | - | x | - | - | x | - | - | x | - | x | - | - | - | x | - | - | - | x | 6 | + |
| Zgheib (41) | 2010 | x | - | - | x | - | x | - | - | x | - | x | - | x | - | - | - | - | x | - | - | - | x | 7 | + |
| Henkin (36) | 2003 | x | - | - | x | - | - | x | - | x | - | x | x | - | x | - | - | - | x | - | x | - | - | 8 | + |
| Matsumura (32) | 2013 | x | - | - | x | - | x | - | - | x | - | x | x | - | x | - | - | - | x | - | - | - | x | 8 | + |
| Oliveira (39) | 2009 | x | - | - | x | - | x | - | - | x | - | x | x | - | - | x | - | - | x | - | x | - | - | 8 | + |
| Taşkin (20) | 2006 | x | - | - | x | - | x | - | - | x | - | x | x | x | - | - | - | - | x | - | x | - | - | 9 | + |

1: case definition (a – yes with independent validation; b – yes with record linkage, self report, non-independent validation; c – no description), 2: representativeness of cases (a – consecutive or obviously representative series of cases; b – potential selection bias or not stated), 3: selection of control (a – community controls; b – hospital controls; c – no description), 4: definition of control (a – no history of disease/endpoint; b – no description), 5: comparability/adjusting for confounders (a – controls for age; b – controls for other additional factors), 6: ascertainment of exposure (a – secure record or two blinded investigators; b – structured interview where blinded to case/control status or one blinded investigator; c – not blinded; d – written self report or medical record only; e – no description), 7: same method of ascertainment of exposure for cases and controls (a – yes; b – no), 8: non response rates (a – same rate for both groups; b – non respondents described; c – rate different and no designation), *: stars given for each question; x: answer marked for each question; +: good, ±: moderate
