# Supplementary material for: Is breast arterial calcification associated with coronary artery disease?—A systematic review and meta-analysis
Source: PLoS One. 2020 Jul 28;15(7):e0236598. doi: 10.1371/journal.pone.0236598 (PMC7386618; doi:10.1371/journal.pone.0236598)
Supplement: S4 Table — 1: representativeness of the sample (a—truly representative of the average target population; b—somewhat representative of the average target population; c—selected group of participants; d—no description); 2: sample size (a—justified and satisfactory including sample size calculation; b—not justified or no information), 3: Ascertainment of risk factor (a—secured record/clinical registry and validated measurement tool used; b—structured questionnaire and validated measurement tool; c—self report; d—no description), 4: non-respondents/missing comparable data (a—the characteristics between BAC+/- are comparable and response rate is >50%; b—the characteristics between BAC+/- is not sufficiently comparable and the response rate is <50%; c—no information provided), 5: comparability/control for confounders (a—data/results adjusted for age; b—data/results adjusted for any additional factors), 6: assessment of BAC (a—independent blinded assessment of BAC; b—single blinded assessment of BAC; c—unblinded assessment of BAC/no description, 7: statistical methods (a—statistical test used to analyse the data clearly described, appropriate and measures of association presented include confidence intervals and p values; b—statistical test not appropriate, not described or incomplete), *: stars given for each question; x: answer marked for each question, +: good, ±: moderate. (DOCX) [file pone.0236598.s004.docx]

| **­Author** | **Year** | **Selection** | | | | | | | | | | | | | **Comparability** | | **Outcome** | | | | | **Total** | **Overall** |
| --- | --- | --- | --- | --- | --- | --- | --- | --- | --- | --- | --- | --- | --- | --- | --- | --- | --- | --- | --- | --- | --- | --- | --- |
|  |  | **1** | | | | **2** | | **3** | | | | **4** | | | **5** | | **6** | | | **7** | |  |  |
|  |  | **a**  ***** | **b**  ***** | **c** | **d** | **a**  ***** | **b** | **a**  ****** | **b**  ***** | **c** | **d** | **a**  ***** | **b** | **c** | **a**  ***** | **b**  ***** | **a**  ****** | **b**  ***** | **c** | **a**  ***** | **b** |  |  |
| Dale (II) (21) | 2008 | x | - | - | - | - | x | - | - | x | - | - | x | - | - | - | - | - | x | - | x | 1 | - |
| Voyvoda (63) | 2019 | - | - | x | - | - | x | - | x | - | - | - | x | - | - | - | - | - | x | - | x | 1 | - |
| Zafar (27) | 2013 | - | x | - | - | - | x | - | - | x | - | - | - | x | - | - | - | - | x | - | x | 1 | - |
| Baum (13) | 1980 | - | x | - | - | - | x | - | x | - | - | - | - | x | - | - | - | - | x | - | x | 2 | - |
| Cetin (17) | 2004 | - | x | - | - | - | x | - | - | x | - | - | - | x | x | - | - | - | x | - | x | 2 | - |
| Topal (38) | 2007 | - | - | x | - | - | x | - | - | x | - | x | - | - | - | - | - | x | - | - | x | 2 | - |
| Moradi (43) | 2014 | - | - | x | - | - | x | - | - | - | x | - | - | x | x | - | - | x | - | - | x | 2 | - |
| Moshyedi (35) | 1995 | - | - | x | - | - | x | x | - | - | - | - | x | - | - | - | - | - | x | - | x | 2 | - |
| Pecchi (55) | 2003 | - | - | x | - | - | x | - | - | x | - | x | - | - | x | x | - | - | x | - | x | 3 | - |
| Sickles(14) | 1985 | x | - | - | - | - | x | x | - | - | - | - | - | x | - | - | - | - | x | - | x | 3 | - |
| Akinola (23) | 2011 | - | x | - | - | - | x | - | x | - | - | - | x | - | - | x | - | - | x | x | - | 4 | - |
| Penugonda (40) | 2010 | - | - | x | - | - | x | x | - | - | - | x | - | - | - | - | - | x | - | - | x | 4 | - |
| Soylu (30) | 2018 | x | - | - | - | - | x | - | - | - | x | x | - | - | x | x | - | - | x | x | - | 5 | ± |
| Chadashvili (45) | 2016 | - | x | - | - | - | x | x | - | - | - | - | x | - | - | - | x | - | - | - | x | 5 | ± |
| Fathala (I) (46) | 2017 | - | - | x | - | - | x | x | - | - | - | x | - | - | x | x | - | x | - | - | x | 6 | ± |
| Sarrafzadegann (71) | 2009 | - | - | x | - | - | x | - | x | - | - | x | - | - | x | x | - | x | - | x | - | 6 | ± |
| Yildiz (II) (56) | 2014 | - | x | - | - | - | x | - | x | - | - | x | - | - | x | x | - | - | x | x | - | 6 | ± |
| Fathala (II) (69) | 2018 | - | x | - | - | - | x | x | - | - | - | x | - | - | x | x | - | - | x | - | x | 6 | ± |
| Hekimoğlu (42) | 2012 | - | - | x | - | - | x | x | - | - | - | - | - | x | - | x | x | - | - | x | - | 6 | ± |
| Karm (44) | 2015 | - | - | x | - | - | x | x | - | - | - | - | - | x | x | x | - | x | - | x | - | 6 | ± |
| Mostafavi (66) | 2015 | x | - | - | - | - | x | x | - | - | - | - | - | x | x | x | x | - | - | - | x | 7 | + |
| Parikh (57) | 2019 | x | - | - | - | - | x | x | - | - | - | x | - | - | x | x | - | - | x | x | - | 7 | + |
| Kataoka (50) | 2006 | - | x | - | - | - | x | - | x | - | - | x | - | - | x | - | x | - | - | x | - | 7 | + |
| Nasser (53) | 2014 | - | x | - | - | x | - | - | x | - | - | - | x | - | x | x | - | x | - | x | - | 7 | + |
| Ružičić (48) | 2018 | - | - | x | - | - | x | x | - | - | - | - | - | x | x | x | x | - | - | x | - | 7 | + |
| Maas (I) (19) | 2006 | x | - | - | - | - | x | - | x | - | - | x | - | - | x | - | x | - | - | x | - | 7 | + |
| Friedlander (26) | 2012 | - | - | x | - | - | x | x | - | - | - | x | - | - | x | x | - | x | - | x | - | 7 | + |
| Crystal (16) | 2000 | x | - | - | - | - | x | - | - | x | - | x | - | - | x | x | x | - | - | x | - | 7 | + |
| Van Noord (15) | 1996 | x | - | - | - | - | x | - | x | - | - | - | - | x | x | x | x | - | - | x | - | 7 | + |
| McLenachan (49) | 2019 | - | - | x | - | - | x | x | - | - | - | x | - | - | x | x | x | - | - | x | - | 8 | + |
| Margolies (34) | 2016 | - | x | - | - | - | x | x | - | - | - | x | - | - | x | x | - | x | - | x | - | 8 | + |
| Fiuza Ferreira (37) | 2007 | - | - | x | - | - | x | x | - | - | - | x | - | - | x | x | x | - | - | x | - | 8 | + |
| Ronzani (68) | 2017 | - | - | x | - | x | - | - | x | - | - | x | - | - | x | x | x | - | - | x | - | 8 | + |
| Ferreira (52) | 2009 | - | - | x | - | x | - | x | - | - | - | x | - | - | x | - | x | - | - | x | - | 8 | + |
| Maas (II) (58) | 2004 | - | - | x | - | - | x | x | - | - | - | x | - | - | x | x | x | - | - | x | - | 8 | + |

1: representativeness of the sample (a – truly representative of the average target population; b – somewhat representative of the average target population; c – selected group of participants; d – no description); 2: sample size (a – justified and satisfactory including sample size calculation; b – not justified or no information), 3: Ascertainment of risk factor (a – secured record/clinical registry and validated measurement tool used; b – structured questionnaire and validated measurement tool; c – self report; d – no description), 4: non-respondents/missing comparable data (a – the characteristics between BAC+/- are comparable and response rate is >50%; b – the characteristics between BAC+/- is not sufficiently comparable and the response rate is <50%; c – no information provided), 5: comparability/control for confounders (a – data/results adjusted for age; b – data/results adjusted for any additional factors), 6: assessment of BAC (a – independent blinded assessment of BAC; b – single blinded assessment of BAC; c – unblinded assessment of BAC/no description, 7: statistical methods (a – statistical test used to analyse the data clearly described, appropriate and measures of association presented include confidence intervals and p values; b – statistical test not appropriate, not described or incomplete), *: stars given for each question; x: answer marked for each question, +: good, ±: moderate
