# Supplementary figures and images for: Is breast arterial calcification associated with coronary artery disease?—A systematic review and meta-analysis
Source: PLoS One. 2020 Jul 28;15(7):e0236598. doi: 10.1371/journal.pone.0236598 (PMC7386618; doi:10.1371/journal.pone.0236598)

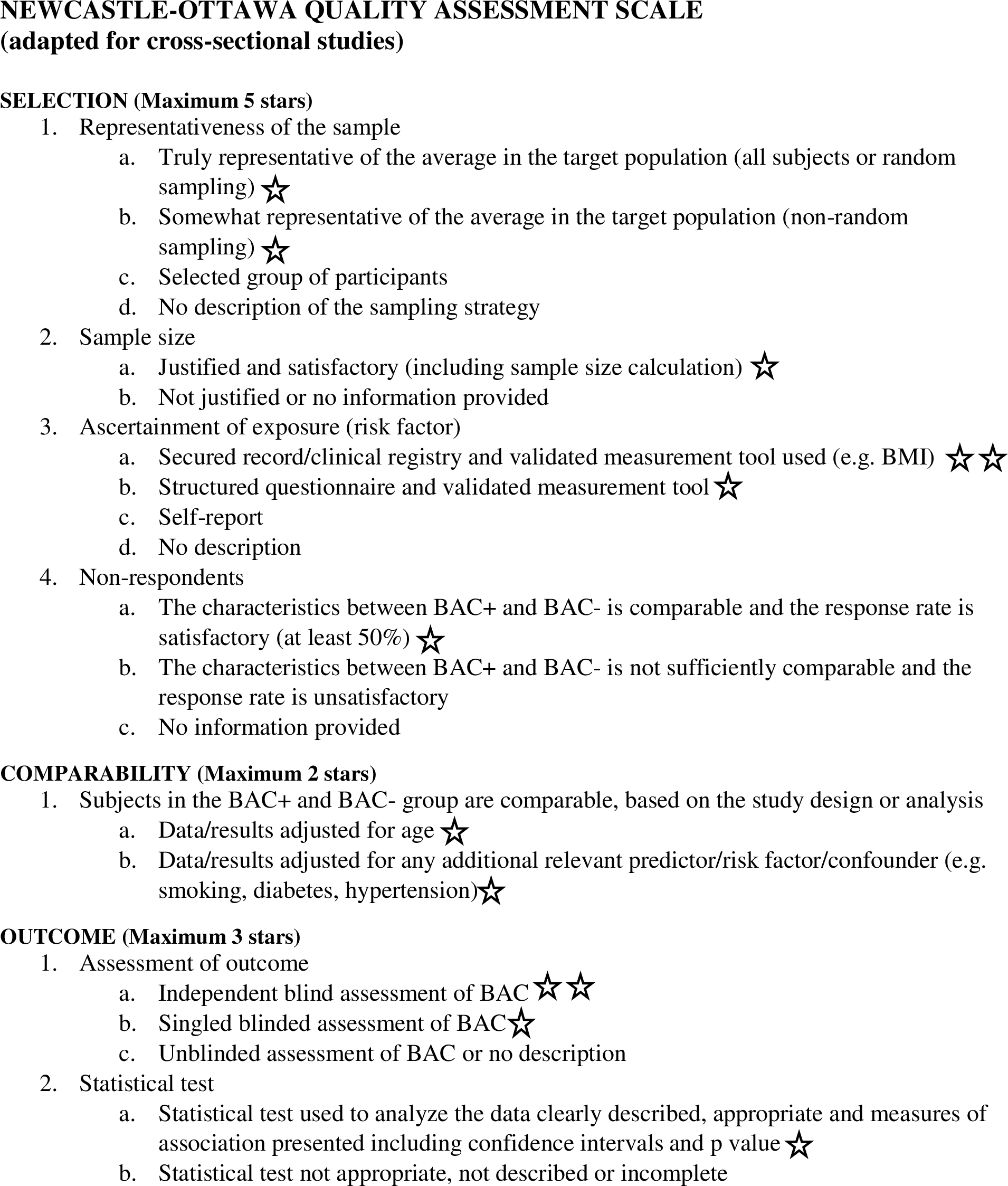

Supplement: S1 Fig — (TIF) [file pone.0236598.s005.tif]

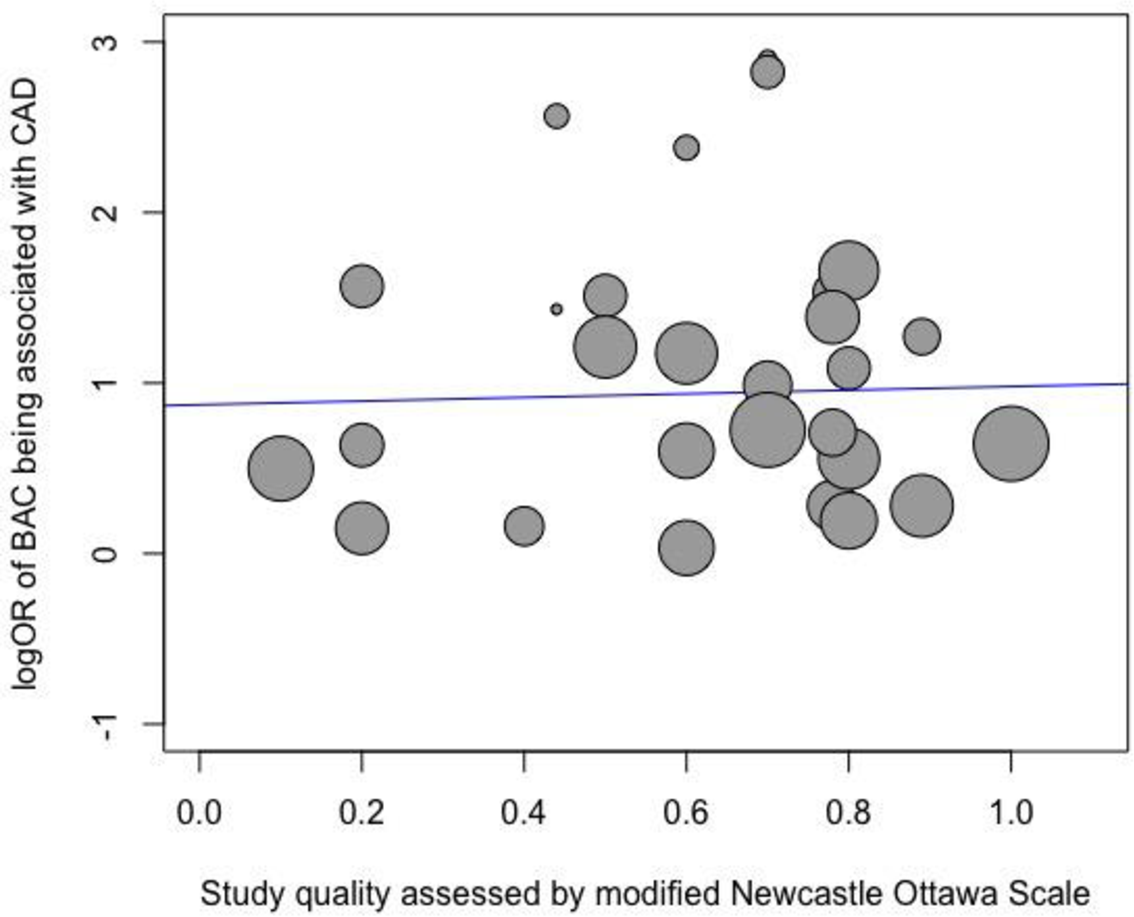

Supplement: S2 Fig — Each bubble represents one single study and its size corresponds to the study sample size. The odds of BAC being associated with CAD had a slight upward trend with quality of the study (slope = 0.059; p = 0.889). (TIF) [file pone.0236598.s006.tif]

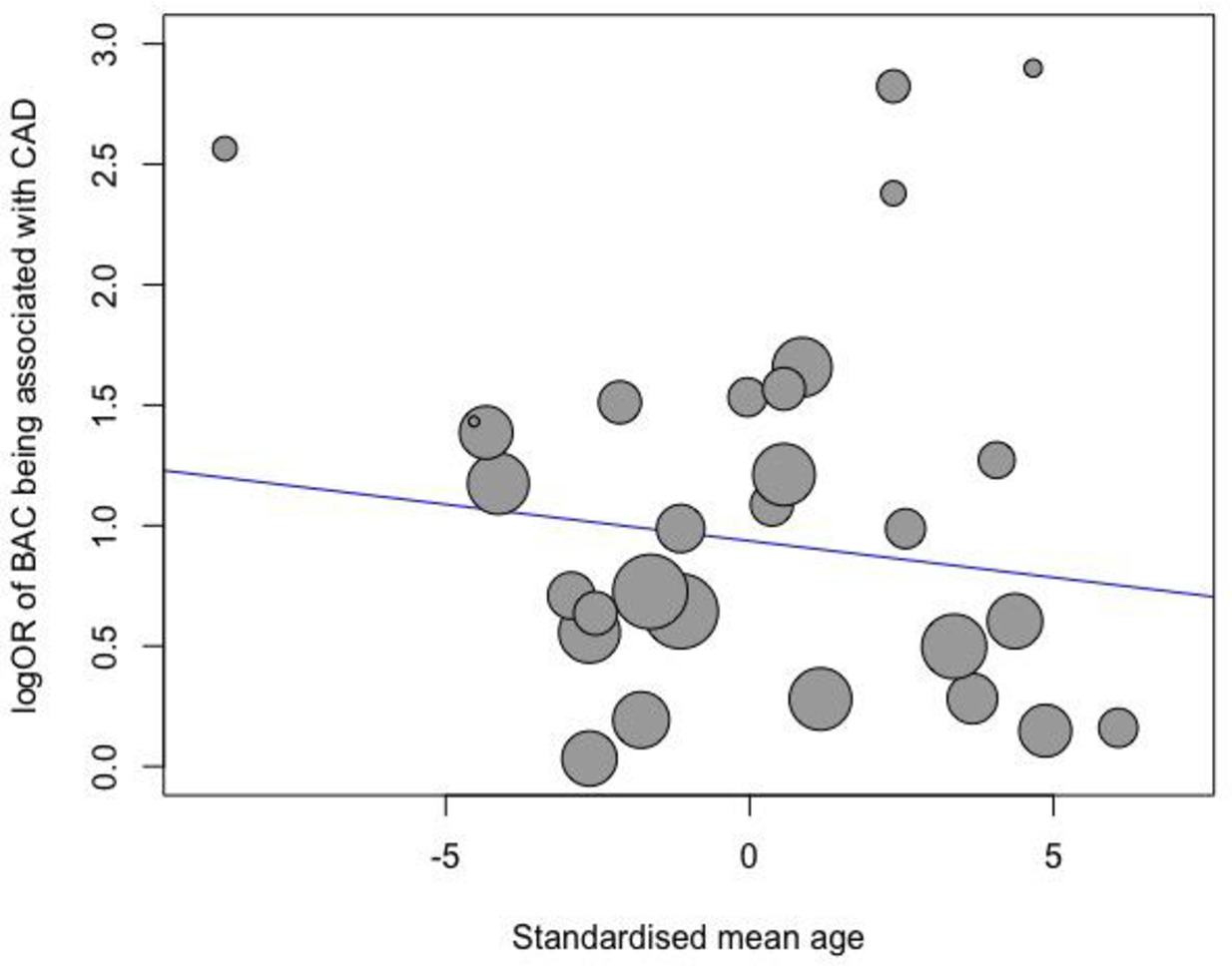

Supplement: S3 Fig — Standardised mean age was calculated as mean age of all study included in analysis—mean age of individual study. Each bubble represents one single study and its size corresponds to the study sample size. The odds of BAC being associated with CAD had a downward trend with age (slope = -0.032, p = 0.323). (TIF) [file pone.0236598.s007.tif]

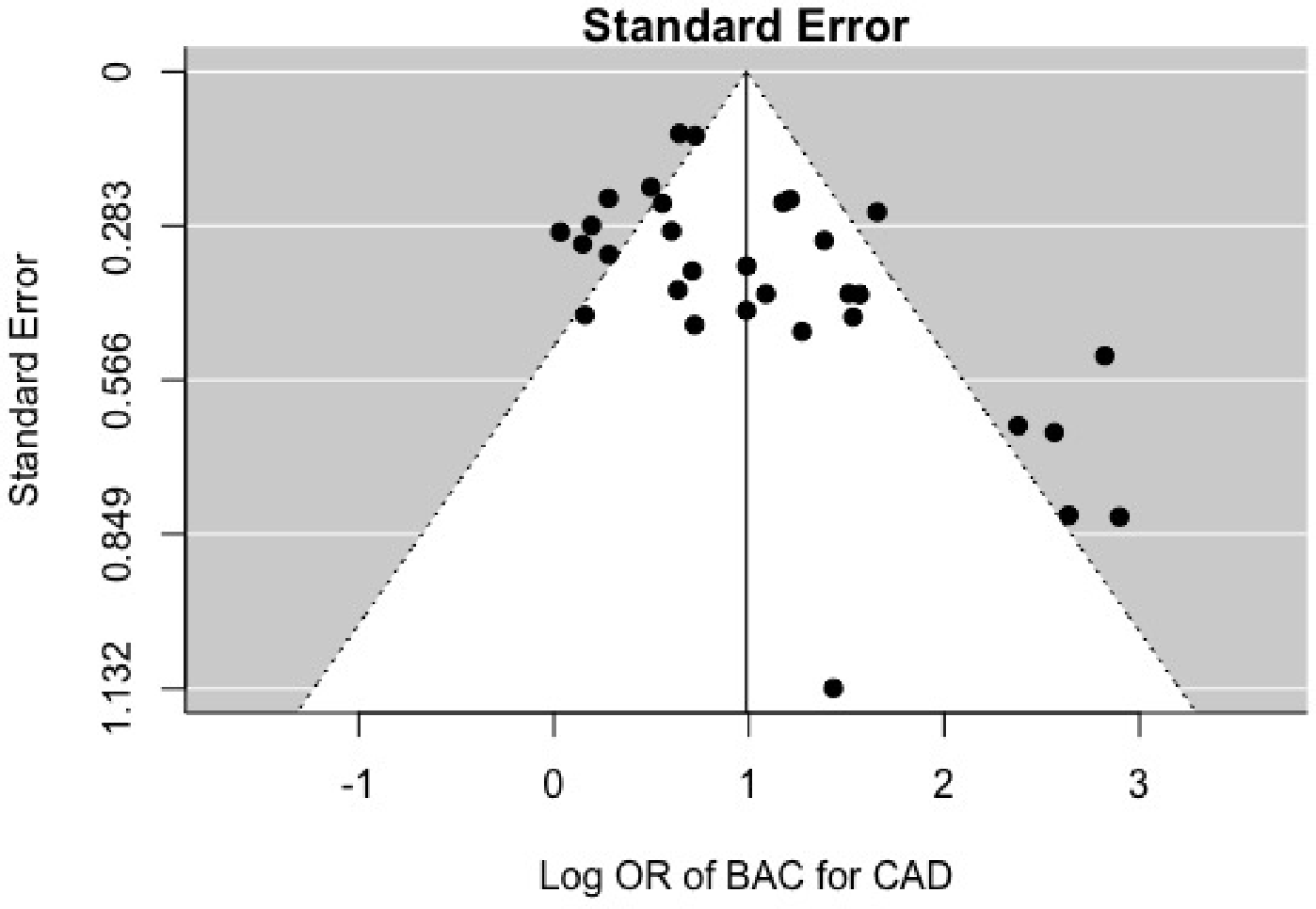

Supplement: S4 Fig — Each dot represents a study. The solid vertical line represents the summary estimate of the log odds ratio of BAC and CAD and the triangle is fixed on the summary estimate and extends 1.96 standard errors either side. The distribution of studies are asymmetrical and more than 5% of the studies are outside the white triangle which indicates potential publication bias but could also result from heterogeneity. (TIF) [file pone.0236598.s008.tif]

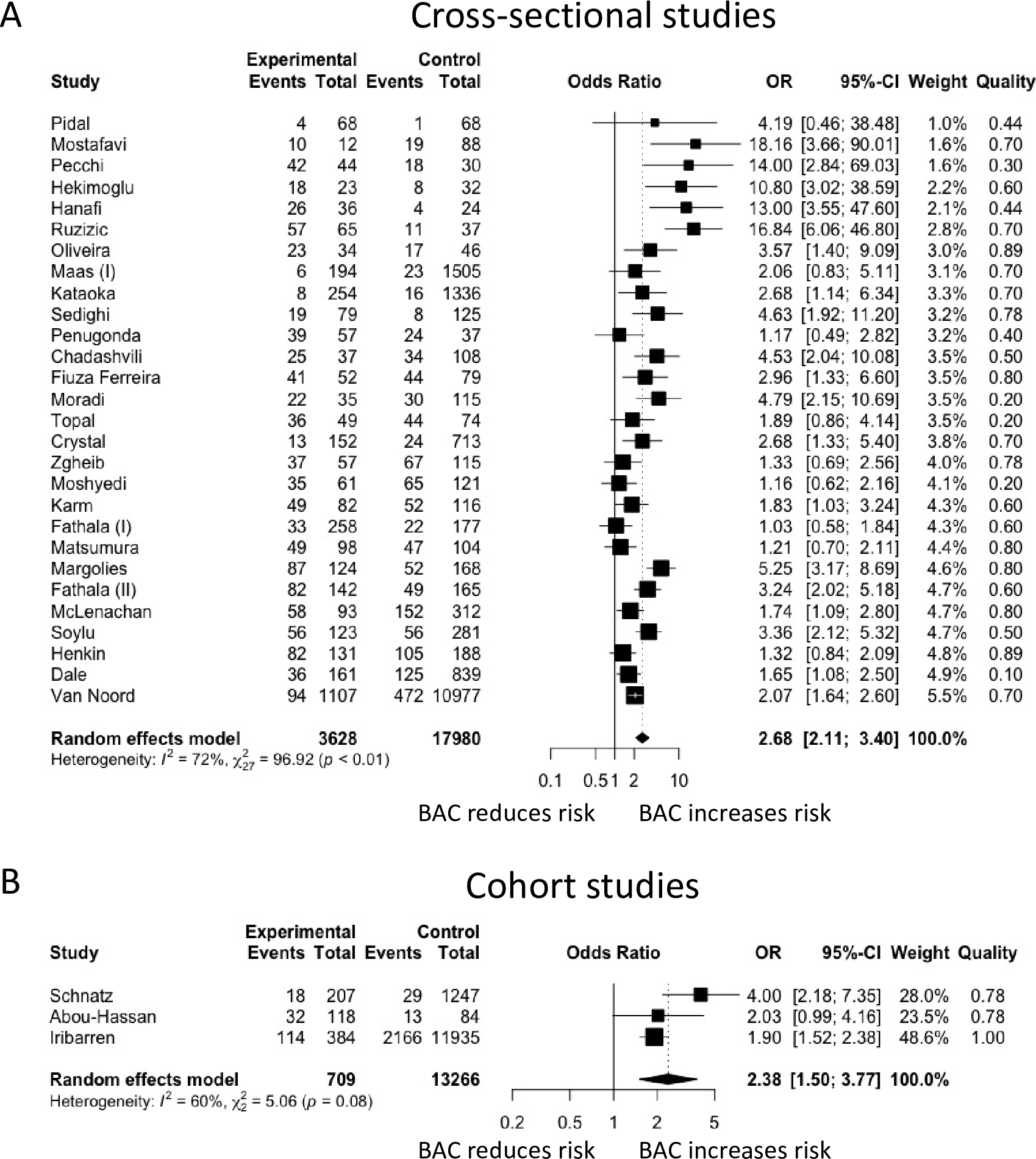

Supplement: S5 Fig — A shows the association of BAC and CAD in cross-sectional studies. S5 Fig B shows the association of BAC and CAD in cohort studies. (TIF) [file pone.0236598.s009.tif]
